# Effects of Sex and Seasonal Climatic Changes on the Risk of Incidence of Anti-EGFR Therapy-Induced Rash in Cancer Patients: A Retrospective Study

**DOI:** 10.3390/medicina57080801

**Published:** 2021-08-04

**Authors:** Takahiro Arai, Yukiyoshi Fujita, Hisao Imai, Hiroe Matsumoto, Miho Yamazaki, Eriko Hiruta, Yuka Suzuki, Hitoshi Ojima, Hisashi Hosaka, Koichi Minato, Taeko Saito

**Affiliations:** 1 Division of Pharmacy, Gunma Prefectural Cancer Center, Ota 373-8550, Gunma, Japan; takahiroarai1210@gunma-cc.jp (T.A.); e-hiruta@gunma-cc.jp (E.H.); aoyu0609@gmail.com (Y.S.); tae.saito@gunma-cc.jp (T.S.); 2 Division of Respiratory Medicine, Gunma Prefectural Cancer Center, Ota 373-8550, Gunma, Japan; m06701014@gunma-u.ac.jp (H.I.); kminato@gunma-cc.jp (K.M.); 3 Department of Respiratory Medicine, Comprehensive Cancer Center, International Medical Center, Saitama Medical University, Hidaka 350-1298, Saitama, Japan; 4 Division of Nursing, Gunma Prefectural Cancer Center, Ota 373-8550, Gunma, Japan; matsumoto.h@gunma-cc.jp (H.M.); miho_ya_ma@yahoo.co.jp (M.Y.); 5 Department of Gastroenterological Surgery, Gunma Prefectural Cancer Center, Ota 373-8550, Gunma, Japan; hiojima@gunma-cc.jp; 6 Division of Gastroenterological Medicine, Gunma Prefectural Cancer Center, Ota 373-8550, Gunma, Japan; hosaka@gunma-cc.jp

**Keywords:** colorectal cancer, head and neck cancer, rash, seasonal variations, anti-EGFR monoclonal antibody, sexual difference

## Abstract

*Background and Objectives*: Seasonal climatic changes may affect the development of the rash that is characteristic of treatment with anti-epidermal growth factor receptor (EGFR) antibodies. We evaluated the association between seasons and rash incidence among patients with cancer. *Materials and Methods*: Data from patients with colorectal or head and neck cancer treated with cetuximab or panitumumab during summer (S group; *n* = 34) or winter (W group; *n* = 37) between June 2014 and February 2019 were collected to retrospectively examine patient characteristics and rash incidence ≤ 8 weeks after treatment initiation. *Results:* Rashes were observed in 73.5% (*n* = 25) and 78.4% (*n* = 29) and grade 3 rashes were observed in 17.6% (*n* = 6) and 2.7% (*n* = 1) of the patients in the S and W groups, respectively. The incidence of grade ≥ 2 rashes in males in the S group was higher than that in the rest of the patient groups (*p* < 0.01). *Conclusions:* The higher incidence of skin rashes in males during summer might be attributed to the effects of ultraviolet light, lack of skincare, male hormones, and secretion of anti-EGFR antibodies in sweat. These findings highlight the need for research on preventive measures for such rashes.

## 1. Introduction

Skin toxicity is one of the common adverse effects of cancer chemotherapy and is manifested in several ways, such as erythema over palmoplantar surfaces, rash, dry skin, and pruritus. Although skin toxicity is not life-threatening, it affects daily life in severe cases and leads to dose reduction or cancer chemotherapy discontinuation [1].

Skin rash is frequently associated with the administration of epidermal growth factor receptor (EGFR) inhibitors, including tyrosine kinase inhibitors, such as gefitinib and erlotinib, and anti-EGFR monoclonal antibodies, such as cetuximab and panitumumab [2]. EGFR tyrosine kinase inhibitors are used mainly to treat lung cancer. In contrast, anti-EGFR monoclonal antibodies are essential drugs used for colorectal cancer (CRC) and head and neck cancer (HNC). Prophylactic treatment using skin moisturizers, sunscreens, topical steroids, and tetracycline antibiotics can reduce the incidence of skin toxicity due to exposure to anti-EGFR monoclonal antibodies [3,4]. Rash severity has been positively correlated with the clinical outcomes of EGFR inhibitor administration in several cancer types [5,6]. Therefore, it is critical to optimize the prophylactic management of skin toxicity induced by such treatments.

**Figure 1:**
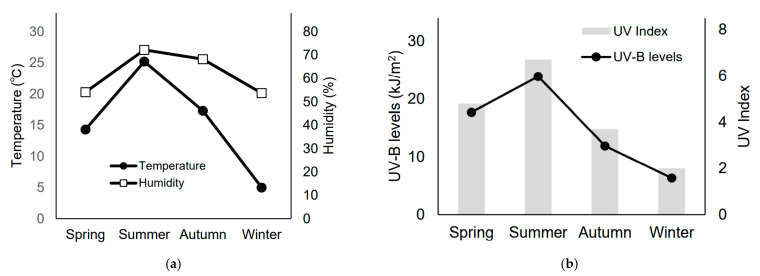
Observational data in different Japanese seasons. (**a**) The average ambient temperature (°C; left axis) and average humidity (%; right axis) (Maebashi, 2014–2019). Modified from “Historical weather data” with permission from the Japan Meteorological Agency. (**b**) The average ultraviolet (UV)-B levels (kJ/m^2^; left axis) and average UV index (right axis) (Tsukuba, 2014–2019). Modified from “UV data collection” with permission from the Japan Meteorological Agency.

As defined by the Japan Meteorological Agency, the four seasons in Japan are Spring (March to May), Summer (June to August), Autumn (September to November), and Winter (December to February) [7]. Various climatic changes characterize the four seasons. For instance, in Maebashi City, Gunma Prefecture, the average temperature and humidity from 2014 to 2019 were 25.2 °C and 72.2% in summer and 5.0 °C and 53.7% in winter, respectively. The changes in temperature and humidity for each season according to the Japan Meteorological Agency are shown in Figure 1a [8]. Owing to these climatic changes, in Japan, the water content of the skin′s stratum corneum in healthy subjects has been reported to decrease significantly in winter compared to that in summer [9]. Furthermore, atopic dermatitis symptoms are worse during winter [10]. These seasonal changes are considered to be a result of low-humidity and low-temperature environments and decreased skin barrier function [11]. Therefore, it may be hypothesized that skin toxicity due to cancer chemotherapy may vary on the basis of season and sex. For instance, a previous report that studied the relationship between skin toxicities and seasons showed that the incidence of a hand–foot syndrome caused by sunitinib was higher in summer than in other seasons [12]. Furthermore, males have been reported to be at a greater risk of severe cetuximab-induced rash [13]. However, the relationship between EGFR inhibitor-induced rash and season or sex has not been investigated.

This study aimed to analyze the relationship between rash incidence and seasonal changes and sex in patients with CRC or HNC treated with anti-EGFR monoclonal antibodies.

## 2. Methods

### 2.1. Season Definition and Patients

This study retrospectively reviewed the cohort of consecutive patients who received cetuximab or panitumumab for CRC or HNC, during summer or winter, between 1 June 2014 and 28 February 2019. Cetuximab (500 mg/m^2^) or panitumumab (6 mg/kg) were administered every 2 weeks in patients with CRC. Cetuximab was administered at an initial loading dose of 400 mg/m^2^ and a maintenance dose of 250 mg/m^2^ once a week in patients with HNC. On receiving treatments, patients were instructed by pharmacists on the need to moisturize, protect, and cleanse the skin. When the treatment was continued, a nurse checked the patient′s skin condition and explained the importance of skin care to the patients. Topical steroids were used to treat rashes (hydrocortisone butyrate 0.1% was applied to the face, and difluprednate ointment 0.05% was applied to the rest of the body). Patients who received treatment during summer were assigned to the S group, and those who were treated in winter were assigned to the W group. Summer was defined as the period from June to August, and winter was the period from December to February. Patients who received EGFR inhibitors within 1 year prior after the start of treatment with cetuximab or panitumumab, who discontinued treatment with anti-EGFR antibodies within two courses, and/or who received regular oral steroids prior to treatment were excluded from the study.

### 2.2. Evaluation Method

We retrospectively examined the sex, age, body surface area, treatment dose, Eastern Cooperative Oncology Group performance status, and rash incidence of all patients, using data extracted from their medical information records. The primary endpoint was the incidence of a skin rash. The incidence and severity of rash were evaluated up to 8 weeks after the start of treatment with anti-EGFR antibodies. The severity of the rash during every treatment cycle was evaluated based on the Common Terminology Criteria for Adverse Events (CTCAE), version 4.0 (Table A1). The severity of the rash was determined using patient medical records and medical staff evaluations.

### 2.3. Statistical Analysis

Continuous variables were presented as medians (ranges), and categorical variables as percentages (%). The continuous variables were compared using the Mann–Whitney U test, and those between multiple groups were compared using the Kruskal–Wallis test. Categorical variables were compared using the χ^2^ test. When expected cell counts were found to be less than five in the contingency table, Fisher′s exact test with Yate′s continuity correction was used. Time-to-events was analyzed using the Kaplan–Meier method and compared using the log-rank test. Hazard ratios were calculated using a Cox proportional hazard model. All analyses were performed using StatMate V (Atoms, Tokyo, Japan). A two-tailed *p*-value < 0.05 was considered indicative of statistical significance. Based on previous studies, we assumed that the incidence of all grades of rash would be 70% in summer and 35% in winter [4,8,10]. Sixty-two patients were required to detect a difference in rash incidence between the groups with a power of 80% and a significance value of 0.05. Assuming that some data would be missing in 10% of the patients, a sample size of 69 was determined.

## 3. Results

### 3.1. Patient Characteristics

**Figure 2:**
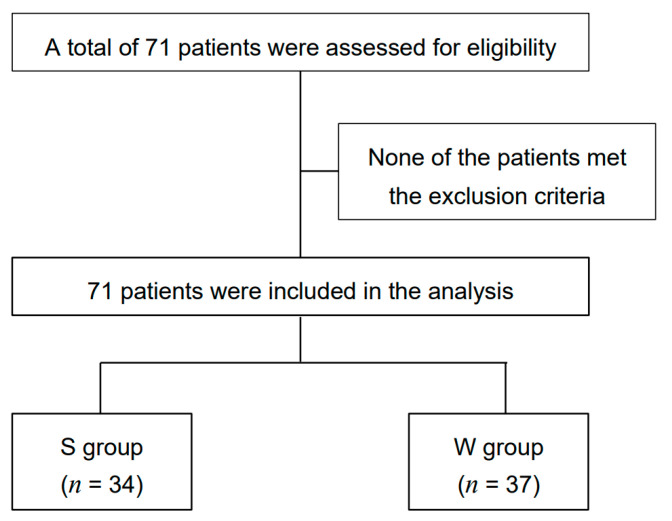
Consolidated Standards of Reporting Trials diagram for the study. S group, patients who received treatment in summer (June to August); W group, patients who received treatment in winter (December to February).

We identified 71 patients treated with cetuximab or panitumumab for CRC or HNC during the summer (*n* = 34) and winter (*n* = 37) seasons, and these patients were assigned to the S and W groups, respectively. None of these patients met the exclusion criteria (Figure 2). The characteristics of the patients in both groups were comparable (Table 1).

### 3.2. Seasonal Differences in Skin Toxicities

**Figure 3:**
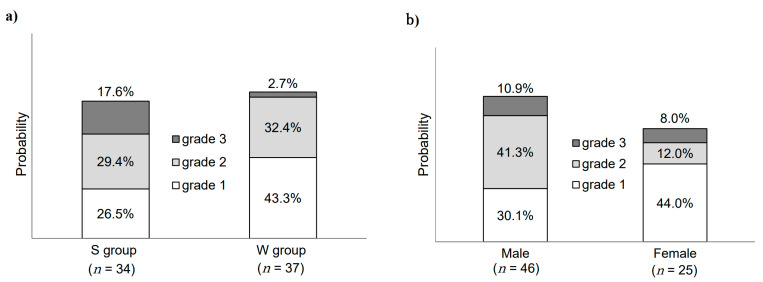
Incidence of acne-like rashes in patients with cancer treated with either cetuximab or panitumumab. (**a**) Incidence of acne-like rashes during the summer (S) and winter (W). (**b**) Incidence of acne-like rashes in males and females.

**Figure 4:**
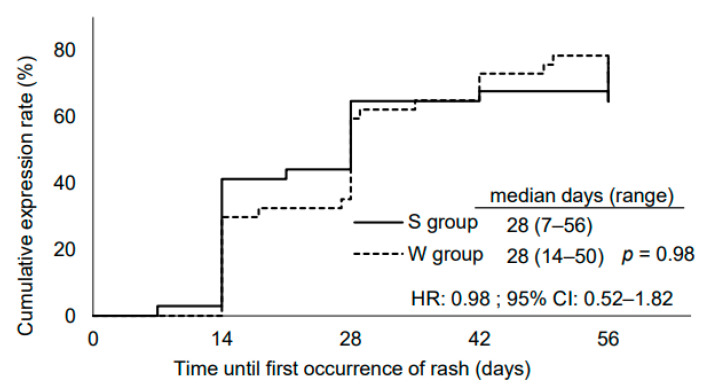
Distribution of time until the first incidence of rash. HR, hazard ratio; CI, confidence interval.

All grades of rashes were observed in 73.5% (*n* = 25) and 78.4% (*n* = 29) of the patients in the S and W groups, respectively; however, no significant difference was observed between the two groups (*p* = 0.63) (Figure 3a). Grade 3 rash was present in 17.6% (*n* = 6) and 2.7% (*n* = 1) of the patients in the S and W groups, respectively, indicating increased frequency during summer; however, the intergroup difference was not significant (*p* = 0.09) (Figure 3a). In addition, the Kaplan–Meier analysis indicated that the median number of days for the incidence of rash was the same (28 days) in both groups (hazard ratio: 0.98; 95% confidence interval (CI): 0.52–1.82; *p* = 0.98) (Figure 4).

### 3.3. Sex-Related Differences in Skin Toxicities

**Figure 5:**
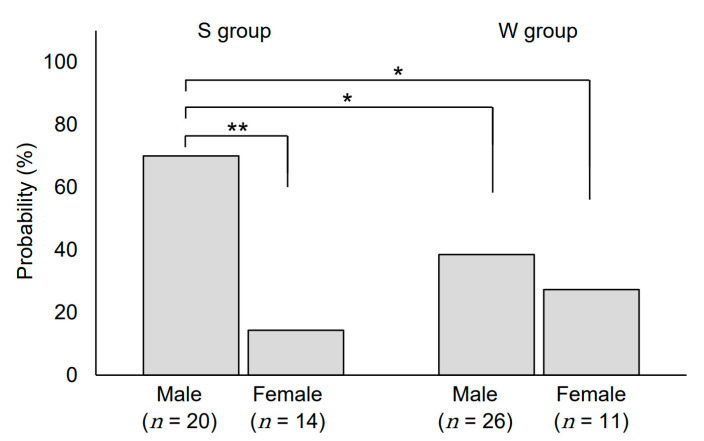
Incidence of grade 2 or higher acne-like rashes based on sex and season. Left, summer (S); right, winter (W). * *p* < 0.05, ** *p* < 0.01.

All grades and grade ≥ 2 rashes were observed in 81.3% (*n* = 38) and 52.2% (*n* = 24) of males, and 64.0% (*n* = 16) and 20.0% (*n* = 6) of females, respectively, with a significant difference in rash severity between the two groups (*p* = 0.02) (Figure 3b). In addition, we analyzed the association between sex and frequency of rashes in both groups. Our analysis showed that the frequency of grade ≥ 2 rashes was significantly higher in males in group S than in any other group (*p* < 0.01) (Figure 5). However, there was no significant difference with respect to the rash incidence frequency between males and females in group W (Figure 5). Incidentally, there was no significant difference in RDI between the four groups of males and females in summer and in winter (*p* = 0.57).

## 4. Discussion

Our results did not indicate any significant differences in the incidence of rashes upon treatment with anti-EGFR antibodies between the S and W groups. Although a sex-based evaluation suggested that males were significantly more prone to skin rash than females in summer, there was no significant association between the incidence of rash and sex of the patients treated in winter. The degree of influence of seasonal changes in the incidence of rashes may differ between males and females. Therefore, when males and females were combinedly considered in the analysis, there may have been no difference between the S and W groups. The tissue concentrations of EGF and EGF receptors are regulated by sex hormones such as estrogen and testosterone, which may contribute to sex differences in the development of skin rashes induced by anti-EGFR antibodies [14]. The higher incidence of skin rashes in males during summer may be attributed to the effects of ultraviolet (UV) light, lack of skincare, male hormones, and secretion of anti-EGFR antibodies into the sweat.

UV rays are classified as UV-A, UV-B, and UV-C, based on their wavelengths. When the skin is exposed to UV-B radiation, photochemical reactions form cyclobutane-type pyrimidine dimers and (6-4)-adducts, which induce DNA damage and inflammatory reactions, thus decreasing epidermal barrier function [15,16]. Reduced epidermal barrier function leads to increased sensitivity to UV-B, making erythema more likely to develop, even at low UV-B doses [17]. Moreover, the average UV-B levels and UV index in Japan (Tsukuba, 2014–2019) were reported to be 23.9 kJ/m^2^ and 6.7, respectively, in summer and 6.3 kJ/m^2^ and 2.0, respectively, in winter (Figure 1b) [18]. Therefore, summer had higher UV-B levels and UV index than did winter. The UV index is formulated using the International Commission on Illumination reference action spectrum for UV-induced erythema on the human skin. For the average person, a UV index of 0 to 2 means low danger from the sun′s UV rays. Moreover, a UV index of 6 to 7 means a high risk of harm from unprotected sun exposure [19]. The use of sunscreen can prevent erythema, DNA photodamage, and contact hypersensitivity [20]. Additionally, the application of sunscreen and general skincare products is significantly lower in males than in females [21,22].

Epithelial cells of sebaceous glands express androgen receptors, and sebaceous glands are target tissues for androgens [23]. Hence, androgenic species have been reported to interfere with the accumulation of structural proteins that rebuild damaged skin [24]. Furthermore, testosterone levels in males vary with season, with testosterone levels being lower in colder months than in hotter months [25]. Moreover, plasma testosterone levels in males are higher than in females [26].

Based on the abovementioned findings, we hypothesized that factors such as increased UV radiation, lack of skincare, and high testosterone levels in summer could be responsible for the higher incidence of rashes in males than in females in summer. If these hypotheses are valid, skincare during the summer months is essential. Thus, focused patient education on the importance of skincare, particularly in the summer, may reduce rash severity in patients, especially males, with CRC or HNC being treated with anti-EGFR antibodies. 

Our retrospective study had several limitations. First, we could not investigate adherence to topical moisturizers and oral antibiotics, such as minocycline. However, we believe that patient adherence was acceptable, as we repeatedly explained the need for moisturizers and oral antibiotics at the start and continuation of treatment with anti-EGFR monoclonal antibodies. Second, there was a lack of information on the skin moisture content, sunscreen use, and testosterone levels of the included patients. Third, skin rashes other than those induced by anti-EGFR antibodies may also be affected by seasonal changes, but there is a lack of this information. Fourth, this study enrolled patients from a single institution, which may make it difficult to generalize the findings. However, it is important to study the effects of UV radiation on skin by climatic variations, location, and racial differences. Though our retrospective study examined as many factors as possible given the available resources, additional studies that consider the factors of adherence, skin moisture content, sunscreen use, and testosterone levels are needed to expand upon the findings of our study.

The novelty of our study is that we identified no difference in the incidence of rashes caused by treatment with anti-EGFR antibodies between the summer and winter months. However, males during the summer may be more prone to developing rashes than females during the summer as well as males and females during the winter.

## 5. Conclusions

The topic of our study is relevant to the current situation, as an increasing number of patients are receiving anti-EGFR therapy, making them susceptible to rashes. Rashes tend to be more frequent in males during summer. This information should help healthcare staff and patients take the necessary actions, such as education on the practice of moisturizing, sunscreen use, and skin cleansing. However, rash mechanisms and preventive methods still remain unclear. We anticipate that the findings of our study on the relationship between rash and seasonal climate change will assist in the elucidation of rash mechanisms and the establishment of preventive measures.

## Figures and Tables

**Table 1:** Patient characteristics.

	S Group (*n* = 34)		W Group (*n* = 37)		*p*-Value
	No.	%	No.	%	
Sex Male Female	20 14	58.8 41.2	26 11	70.3 29.7	0.32
Type of cancer Colorectal cancer Head and neck cancer	27 7	79.4 20.6	31 6	83.8 16.2	0.87
Therapy regimen Cetuximab-based regimen Panitumumab-based regimen	20 14	58.8 41.2	23 14	62.2 37.8	0.77
Moisturizer prescription Yes No	34 0	100.0 0.0	37	100.0 0.0	-
Minocycline prescription Yes No	33 1	97.1 2.9	36 1	97.3 2.7	0.51
History of allergies Yes No	4 30	11.8 88.2	6 31	16.2 83.8	0.84
	Median (range)		Median (range)		
Age (years)	65 (35–78)		64 (35–78)		0.45
RDI of anti-EGFR mAb	0.73 (0.30–1.00)		0.74 (0.39–1.07)		0.80

S group, patients who received treatment in summer (June to August); W group, patients who received treatment in winter (December to February); RDI, relative dose intensity; anti-EGFR mAb: anti-EGFR monoclonal antibody (cetuximab or panitumumab).

## Data Availability

The data presented in this study are available on request from the corresponding author. The data are not publicly available.

## Appendix A

**Table A1:** Classification of the severity of rash acneiform (CTCAE v4.0).

Grade 1	Grade 2	Grade 3	Grade 4	Grade 5
Papules and/or pustules covering <10% BSA, which may or may not be associated with symptoms of pruritus or tenderness	Papules and/or pustules covering 10–30% BSA, which may or may not be associated with symptoms of pruritus or tenderness; associated with psychosocial impact; limiting instrumental ADL	Papules and/or pustules covering >30% BSA, which may or may not be associated with symptoms of pruritus or tenderness; limiting self-care ADL; associated with local superinfection with oral antibiotics indicated	Papules and/or pustules covering any % BSA, which may or may not be associated with symptoms of pruritus or tenderness and are associated with extensive superinfection with IV antibiotics indicated; life-threatening consequences	Death
